# YAGM: a web tool for mining associated genes in yeast based on diverse biological associations

**DOI:** 10.1186/1752-0509-9-S6-S1

**Published:** 2015-12-09

**Authors:** Wei-Sheng Wu, Chung-Ching Wang, Meng-Jhun Jhou, Yu-Cheng Wang

**Affiliations:** 1 Department of Electrical Engineering, National Cheng Kung University, Tainan, Taiwan

## Abstract

**Background:**

Investigating association between genes can be used in understanding the relations of genes in biological processes. STRING and GeneMANIA are two well-known web tools which can provide a list of associated genes of a query gene based on diverse biological associations such as co-expression, co-localization, co-citation and so on. However, the transcriptional regulation association and mutant phenotype association have not been used in these two web tools. Since the comprehensive transcription factor (TF)-gene binding data, TF-gene regulation data and mutant phenotype data are available in yeast, we developed a web tool called YAGM (Yeast Associated Genes Miner) which constructed the transcriptional regulation association, mutant phenotype association and five commonly used biological associations to mine a list of associated genes of a query yeast gene.

**Description:**

In YAGM, we collected seven kinds of datasets including TF-gene binding (TFB) data, TF-gene regulation (TFR) data, mutant phenotype (MP) data, functional annotation (FA) data, physical interaction (PI) data, genetic interaction (GI) data, and literature evidence (LE) data. Then by using the hypergeometric test to calculate the association scores of all gene pairs in yeast, we constructed seven biological associations including two transcriptional regulation associations (TFB association and TFR association), MP association, FA association, PI association, GI association, and LE association. Moreover, the expression profile association from SPELL database was also included in YAGM. When using YAGM, users can input a query gene and choose any possible subsets of the eight biological associations, then a list of associated genes of the query gene will be returned based on the chosen biological associations.

**Conclusions:**

In this study, we presented the YAGM which provides eight biological associations for mining associated genes of a query gene in yeast. Among the eight biological associations constructed in YAGM, three (TFB association, TFR association, and MP association) are novel ones. By comparing the query results of two well-known web tools (STRING and GeneMANIA), we found that YAGM can find out distinct associated genes of a query gene. That is, YAGM can provide alternative candidates of associated genes for biologists to do further experimental investigation. We believe that YAGM will be a useful web tool for yeast biologists. YAGM is available online at http://cosbi3.ee.ncku.edu.tw/yagm/.

## Background

Exploring the association between genes is a crucial issue in the biology study. It helps biologists to discover the relationship of genes. For example, functional annotation association can be used to predict unknown functions of genes [1], expression profile association can be used to predict co-expressed genes [2], and transcriptional regulation association can be used to predict co-regulated genes [3]. Therefore, it would be helpful if there are web tools which can provide a list of associated genes of a query gene based on diverse biological associations.

STRING [4] and GeneMANIA [5] are two well-known web tools which can provide this kind of services. These two tools return a list of associated genes of a query gene based on diverse biological associations derived from neighbourhood, gene fusion, co-occurrence, co-expression, co-localization, co-citation, co-inheritance, genetic interaction, physical interaction, shared protein domains and so on. Although many kinds of biological associations have been used in these two web tools, the transcriptional regulation association and the mutant phenotype association have not been considered yet. Therefore, given a query gene, these two tools cannot provide a list of genes which have similar transcriptional regulatory mechanisms or similar mutant phenotypes to the query gene. Since the comprehensive transcription factor (TF)-gene binding data, TF-gene regulation data and mutant phenotype data are available in yeast, this gives us an opportunity to construct the transcriptional regulation association and the mutant phenotype association in yeast. Moreover, we have many experiences in developing databases and web tools [6-13].

So here we are able to present a web tool called YAGM (Yeast Associated Genes Miner) which constructs eight biological associations to mine a list of associated genes of a query yeast gene. These biological associations include three novel ones (TF binding association, TF regulation association and mutant phenotype association) and five commonly used ones (functional annotation association, physical interaction association, genetic interaction association, literature evidence association, and expression profile association). Depending on the selected biological associations, YAGM can provide a list of genes which have similar bound TFs, similar regulatory TFs, similar mutant phenotypes, similar functions, similar physical interactions, similar genetic interactions, similar literature evidences, or similar expression profiles to the query gene. Moreover, YAGM has a user-friendly search interface and the search results are visualized as network graphs and tables.

## Construction and Contents

### Construction of YAGM

**Figure 1:**
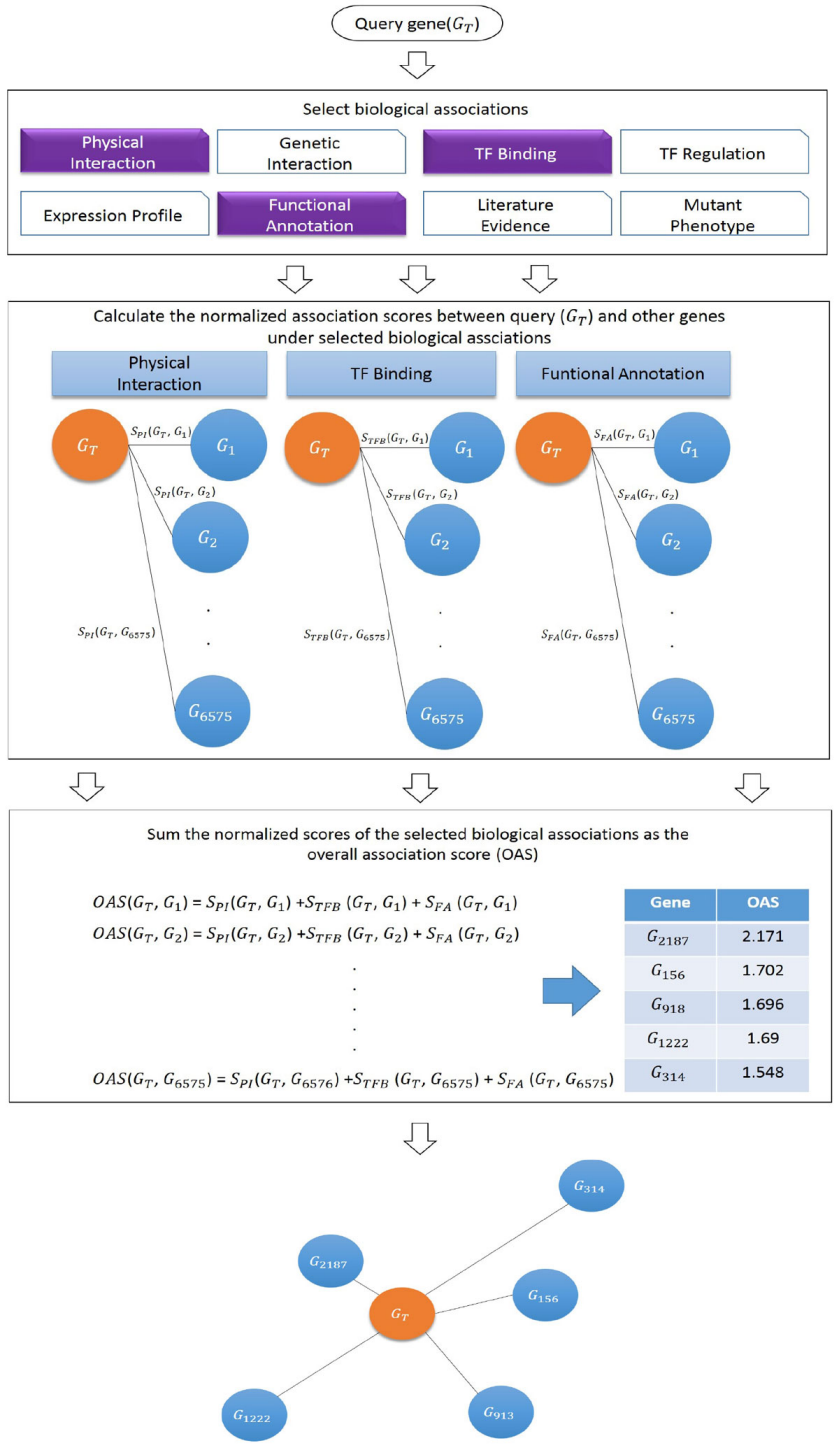
**Construction of YAGM**.

In YAGM, we collected seven kinds of datasets including TF-gene binding (TFB) data, TF-gene regulation (TFR) data, mutant phenotype (MP) data, functional annotation (FA) data, physical interaction (PI) data, genetic interaction (GI) data, and literature evidence (LE) data. Then by using the hypergeometric test to calculate the association scores of all gene pairs in yeast, we constructed seven biological associations including TFB association, TFR association, MP association, FA association, PI association, GI association, and LE association. Moreover, the expression profile (EP) association from SPELL database [2] was also included in YAGM. When using YAGM, users can input a query gene and select any possible subsets of the eight biological associations, then a list of associated genes of the query gene will be returned based on the chosen biological associations (see Figure 1).

### Data collection

Seven kinds of genome-wide datasets were gathered to construct the seven biological associations. First, 41,013 TF-gene binding pairs were retrieved from the YEASTRACT database [14]. Each TF-gene binding pair has experimental evidence (from band-shift, foot-printing or ChIP assay) showing that the TF binds to the promoter of the gene. Second, 168,900 TF-gene regulation pairs were retrieved from the YEASTRACT database. Each TF-gene regulation pair has experimental evidence (from detailed gene by gene analysis or genome-wide expression analysis) showing that the TF perturbation (knockout or over-expression) causes a significant change in the expression of the gene. Third, 605 mutant phenotypes with 10 diverse mutant types were retrieved from SGD database [15]. Fourth, 1,362 yeast functional annotations with 28 main functional categories were retrieved from MIPS database [16]. Fifth, 120,579 physical interactions were retrieved form BioGRID database [17]. Sixth, 190,196 genetic interactions were also retrieved from BioGRID database. Seventh, 70,674 publications associated with genes of interest were downloaded from SGD database.

### Calculation of association scores

A previous study [18] investigated the performance of different association measures (Jaccard index, cosine index, Pearson correlation index and hypergeometric index) in calculating the statistical significance of the overlap of two sets. They found that hypergeometric index performed better than the other indices. Therefore, for each of the seven biological associations, we adopted hypergeometric index, shown in Equation (1), to calculate the association score between the query gene *a *and another gene *b*:

(1) $$H_{i}\left(a,b\right)=-\text{log}\left(\overset{\text{min}\left(m,n\right)}{\underset{x\geq k}{\sum }}\frac{\left(\begin{array}{c} m\\ x \end{array}\right)\left(\begin{array}{c} N-m\\ n-x \end{array}\right)}{\left(\begin{array}{c} N\\ n \end{array}\right)}\right)$$

where *i *= TFB, TFR, MP, FA, PI, GI and LE. The definition of each parameter in Equation (1) is given in Table 1. For example, PI association between genes *a *and *b *measures the significance of the overlap of two sets. The first one is the set of proteins which have physical interactions with the protein product of gene *a *and the second one is the set of proteins which have physical interactions with the protein product of gene *b*.

**Table 1:** Parameters of the Hypergeometic test for the seven constructed biological annotations.

Biological Association	Parameters of the Hypergeometric Test
TF Regulation/TF Binding	N: # of TFs collected from YEASTRACT n: # of TFs that regulate/bind to gene *a* m: # of TFs that regulate/bind to gene *b* k: # of TFs that regulate/bind to both gene *a *and gene *b*

Mutant Phenotype/Literature Evidence	N: # of mutant phenotypes/literature evidences collected from SGD n: # of mutant phenotypes/literature evidences of gene *a* m: # of mutant phenotypes/literature evidences of gene *b* k: # of mutant phenotypes/literature evidences of both gene *a *and gene *b*

Functional Annotation	N: # of functional annotations collected from MIPS n: # of functional annotations of gene *a* m: # of functional annotations of gene *b* k: # of functional annotations of both gene *a *and gene *b*

Physical Interaction/Genetic Interaction	N: # of genes in the yeast genome n: # of genes that have physical/genetic interactions with gene *a* m: # of genes that have physical/genetic interactions with gene *b* k: # of genes that have physical/genetic interactions with both gene *a *and gene *b*

In addition, the expression profile (EP) association H*_EP_*(*a,b*) between the query gene *a *and another gene *b *was retrieved directly from SPELL database [2]. Subsequently, we used Equation (2) to normalize H*_i_*(*a,b*) into the range [0,1] as follows:

(2) $$S_{i}\left(a,b\right)=\frac{H_{i}\left(a,b\right)-\underset{b}{\text{min}}H_{i}\left(a,b\right)}{\underset{b}{\text{max}}H_{i}\left(a,b\right)-\underset{b}{\text{min}}H_{i}\left(a,b\right)}$$

Finally, we summed the normalized scores of the chosen biological associations as the overall association score (*OAS*) between the query gene *a *and another gene *b *shown in Equation (3):

(3) $$OAS\left(a,b\right)=\underset{\begin{array}{c} \;\;t\in chosen\\ \;biological\\ association \end{array}}{\sum }S_{i}\left(a,b\right)$$

### Implementation of the web service of YAGM

The web interface of YAGM is constructed using the PHP language with the CodeIgniter MVC framework. Basic information of yeast genes and scores of eight biological associations for each gene pairs are deposited in MySQL. The table showing the list of associated genes of the query gene is produced by the JQuery. The network graph containing the query gene and all its associated genes is generated by Cytoscape [19].

## Utility and discussion

### Web interface

**Figure 2:**
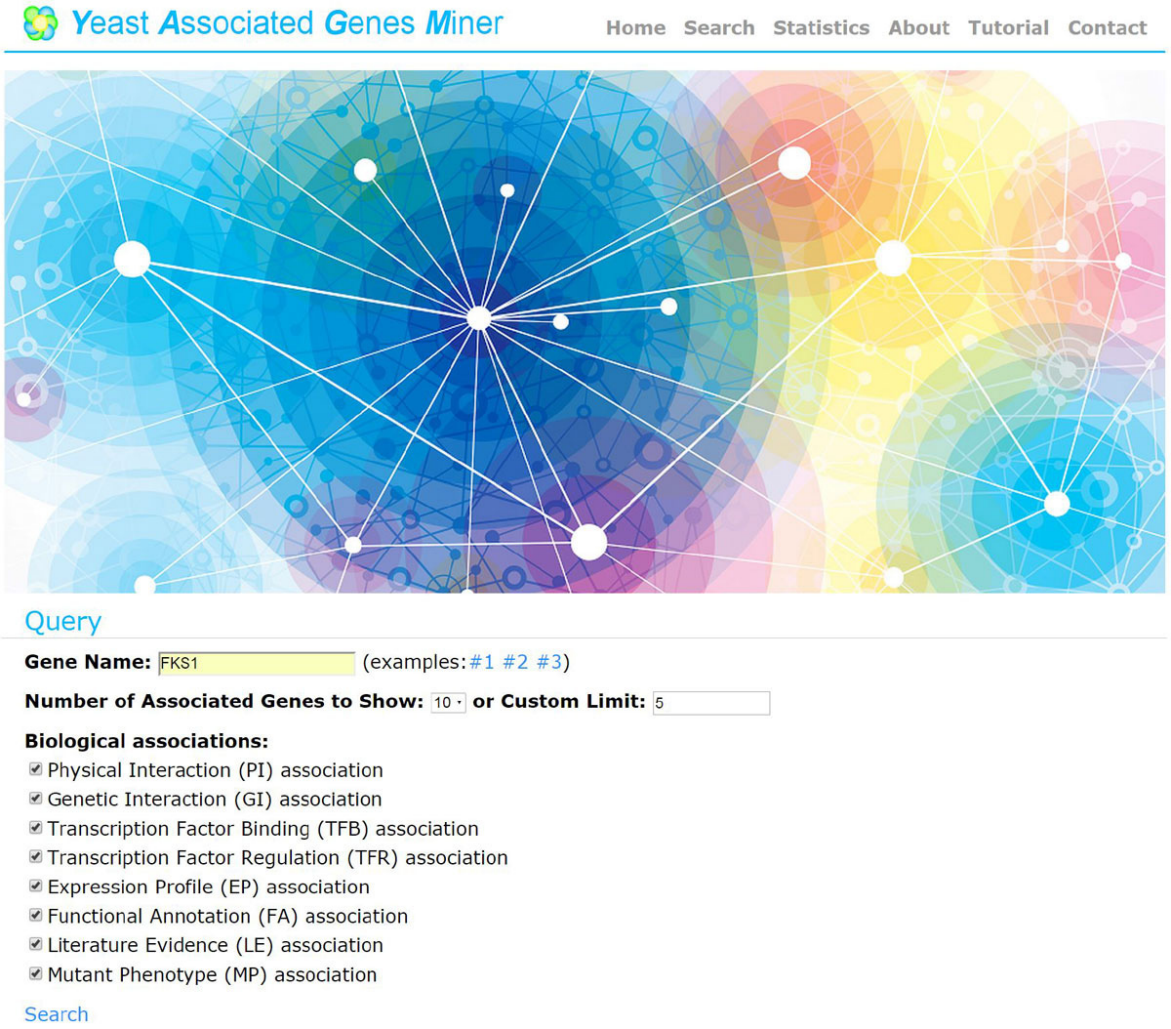
**The query page**. In the query page, users can input a yeast gene name, set the number of associated genes being reported, and select the biological associations being used.

YAGM provides four web pages (the query page, the search result page, the detail page and the reference page) to present the information of a list of associated genes of a query gene based on the selected biological associations. In the query page (Figure 2), users can input a yeast gene name, set the number of associated genes being reported, and select the biological associations being used.

**Figure 3:**
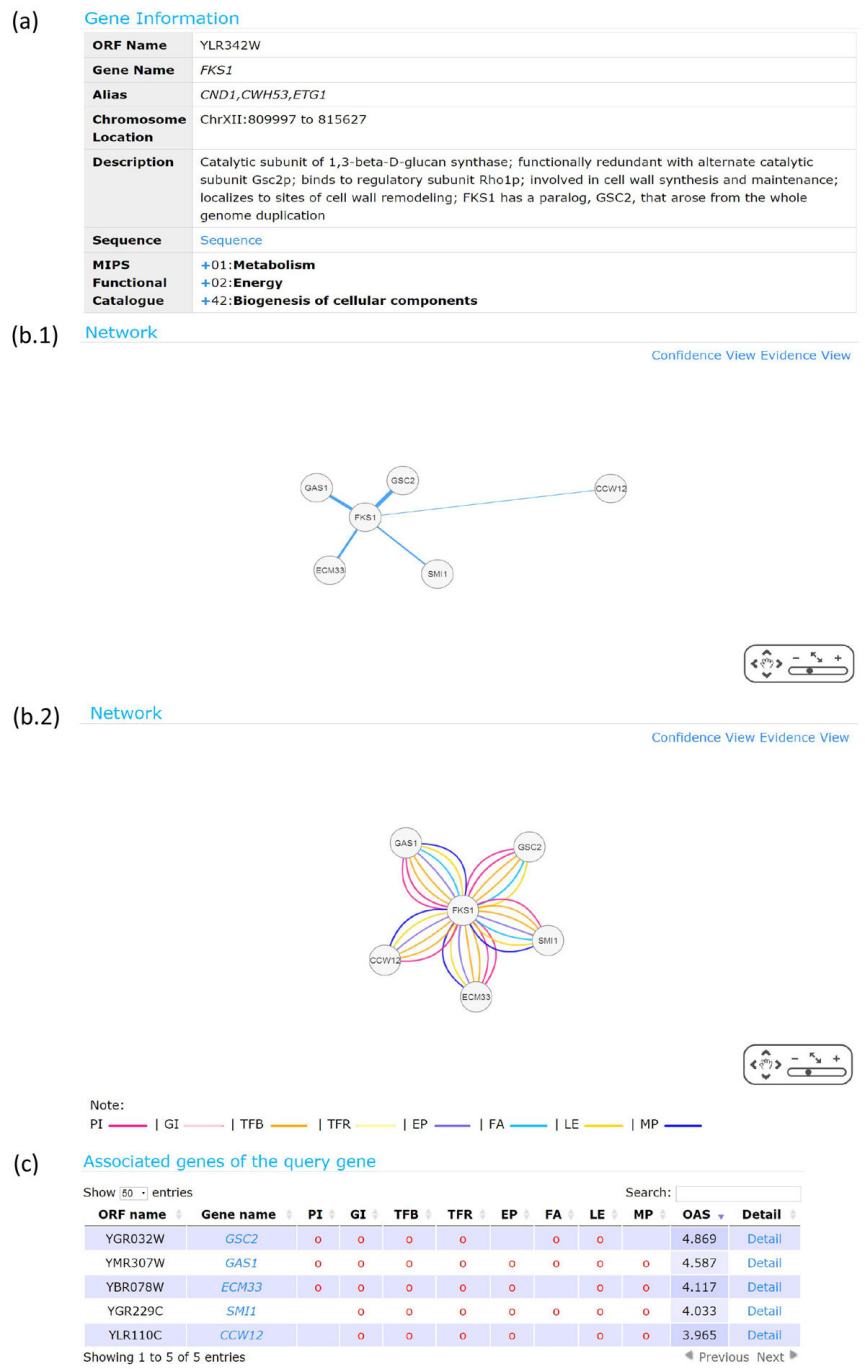
**The search result page**. The search result page can be divided into three parts. (a) The first part contains the basic information (name, chromosome location, description, sequence, and MIPS functional catalogue) of the query gene. (b) The second part contains two network graphs connecting the query gene with all its associated genes. The first network graph is called the confidence view. The edge between the query gene and its associated gene in the network reflects the overall association score (OAS). The higher the OAS, the wider and shorter the edge. The second network graph is called the evidence view. The edge between the query gene and its associated gene in the network indicates that this gene pair has the evidence of a specific biological association. This means that the association score of this gene pair under that biological association is higher than the 95th percentile of the association scores of all gene pairs in the yeast genome. (c) The third part is a table listing the associated genes. In the table, the information of each associated gene contains the evidences of specific biological associations, the OAS, and a link of "Detail".

After submission, users will get a search result page, which can be divided into three parts. The first part (Figure 3a) contains the basic information (name, chromosome location, description, sequence and MIPS functional catalogue) of the query gene. The second part (Figure 3b) contains two network graphs connecting the query gene with all its associated genes. The first network graph is called the confidence view. The edge between the query gene and its associated gene in the network reflects the overall association score (OAS). The higher the OAS, the wider and shorter the edge. The second network graph is called the evidence view. The edge between the query gene and its associated gene in the network indicates that this gene pair has the evidence of a specific biological association. This means that the association score of this gene pair under that biological association is higher than the 95th percentile of the association scores of all gene pairs in the yeast genome. The third part (Figure 3c) is a table listing the associated genes. In the table, the information of each associated gene contains the evidences of specific biological associations, the OAS, and a link of "Detail".

**Figure 4:**
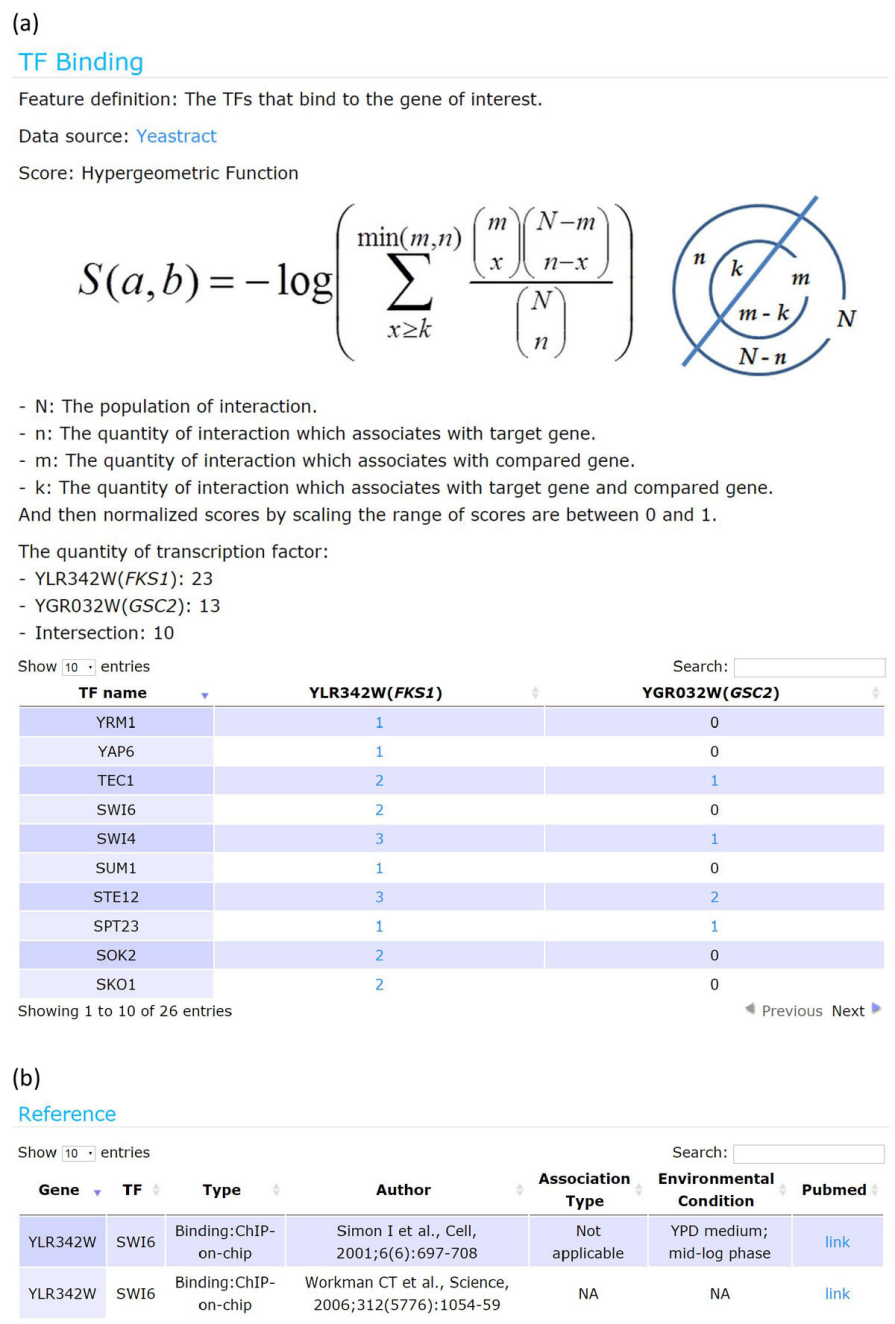
**The detail page and the reference page**. (a) The detail page reveals how the score of each chosen biological association between the query gene and its associated gene is calculated. For example, when calculating the TFB association score between the query gene *FKS1 *and its associated gene *GSC2*, we need to know the list of TFs which bind to *FKS1 *and the list of TFs which bind to *GSC2*. Both lists of TFs are shown in the detail page. (b) The reference page provides the original resource of the data used for calculating the score of a biological association.

When clicking the link of "Detail", users will be directed to the detail page. The detail page (Figure 4a) reveals how the score of each chosen biological association between the query gene and its associated gene is calculated. For example, when calculating the TFB association score between the query gene *FKS1 *and its associated gene *GSC2 *using Equation (1), we need to know the list of TFs which bind to *FKS1*, the list of TFs which bind to *GSC2*, and the list of TFs which bind to both *FKS1 *and *GSC2*. The original resources which provide these three lists of TFs are shown in the reference page (Figure 4b).

### Case study

*FKS1 *is a protein involved in cell wall synthesis and maintenance [15]. Here we input *FKS1 *as a query gene and use all eight biological associations. Then the top five associated genes returned by YAGM is shown in Figure 3c. It can be seen that all these five associated genes have at least six evidences of biological associations, suggesting that they are associated with the query gene in terms of diverse biological associations. We then check the biological plausibility of these five associated gene by using the gene description content in SGD database [15]. Four (*GSC2, GAS1, SMI1, CCW12*) of the five predicted associated genes are known to be involved in cell wall assembly or synthesis just like the query gene *FKS1*, suggesting that YAGM can predict biologically plausible associated genes of a query gene.

### Investigation of the relationships between different biological associations

In order to see how well the different biological associations correlate, for each query gene, we compared the two lists of top 50 associated genes using two different biological associations, respectively. The same process was done for all 6576 possible query genes. Then the average overlap and standard error could be computed (see Additional file 1). We found that the two lists of top 50 associated genes using two different biological associations have low overlap most of the time, indicating different biological associations are usually lowly correlated. The only exception is the TFB-TFR pair. These two biological associations are highly correlated.

Moreover, in order to know which biological associations are more related to the OAS than the others, for each query gene, we compared the two lists of top 50 associated genes using all eight biological associations together and only one biological association, respectively. The same process was done for all 6576 possible query genes. Then the average overlap and standard error could be computed (see Additional file 1). We found that the list of top 50 associated genes using all eight biological associations together have greater average overlap (14 out of 50) with the lists using only TFB association, only TFR association or only LE association than the lists using the other biological associations. This means that TFB association, TFR association and LE association are more informative than the other associations.

### Comparison with related databases

STRING [4] and GeneMANIA [5] are two well-known web tools which can output a list of associated genes of a query gene based on diverse biological associations. Since these two tools provide the same service as our YAGM does, it is informative to do some comparisons. First, we compare the biological associations used in these three tools. As shown in Table 2 four biological associations (physical interaction, genetic interaction, co-expression and co-citation) are commonly used in all three tools, but the others are unique for a particular tool. For example, YAGM has three unique biological associations (TF binding association, TF regulation association and mutant phenotype association). STRING has three unique biological associations (gene fusion evidence, co-occurrence, and pathway evidence). GeneMANIA has three unique biological associations (co-inheritance, co-localization, and shared protein domains).

**Table 2:** Comparison of biological associations constructed in YAGM, STRING and GeneMANIA.

Biological Association\Web tool	YAGM	STRING	GeneMANIA
Physical Interaction	v	v	v

Genetic Interaction	v	v	v

Co-expression	v	v	v

Co-citation	v	v	v

TF Binding	v		

TF Regulation	v		

Mutant Phenotype	v		

Gene Fusion		v	

Co-occurrence		v	

Pathway		v	

Co-inheritance			v

Co-localization			v

Shared Protein Domains			v

Gene Neighbourhoods		v	v

**Figure 5:**
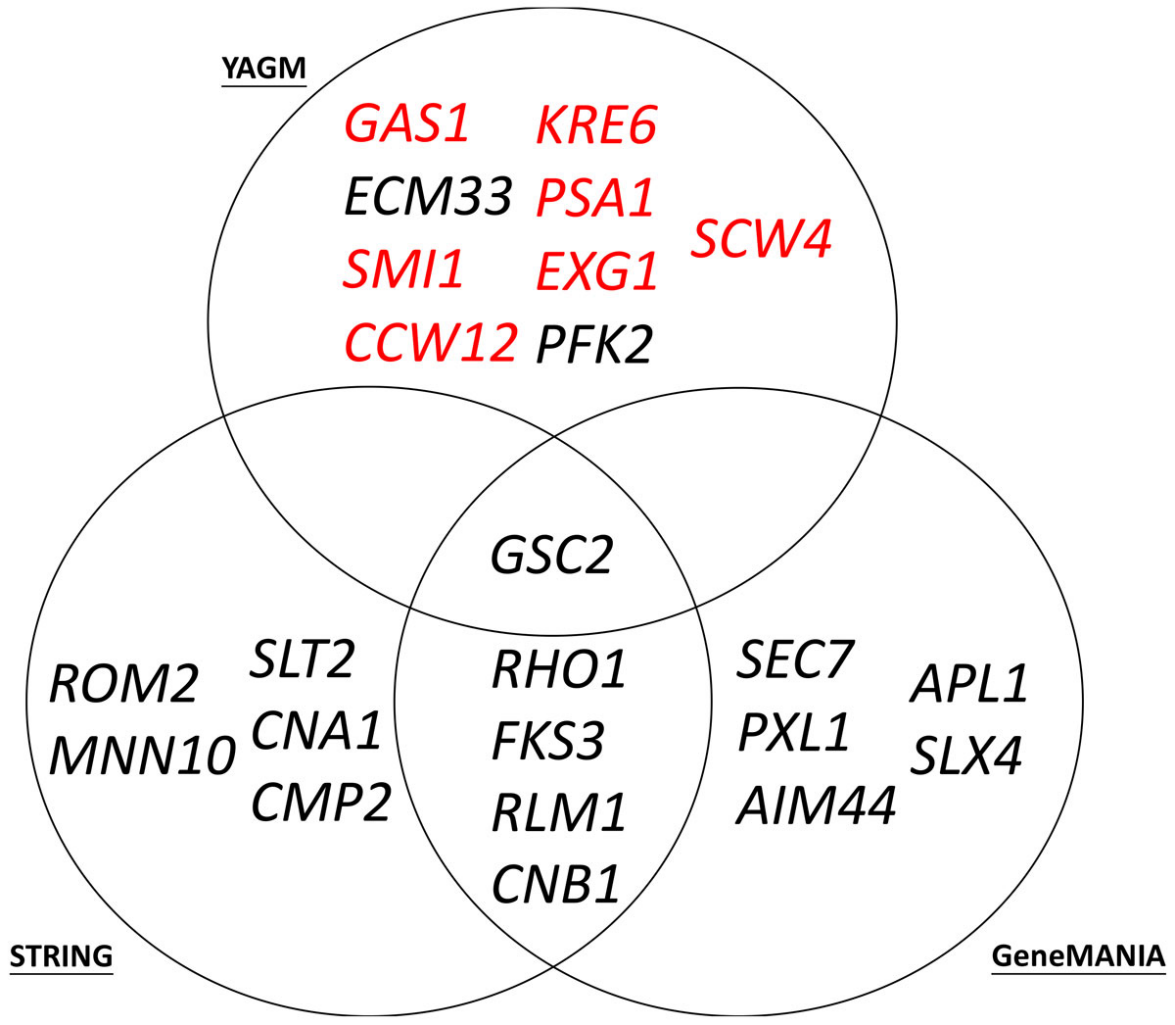
**Comparison of the search results of YAGM, STRING, and GeneMANIA**. Using *FKS1 *as a query gene, we compare the three lists of top ten associated genes obtained from these three tools when all biological associations are used together. It can be seen that nine genes (*GAS1, ECM33, SMI1, CCW12, KRE6, PSA1, EXG1, PFK2 *and *SCW4*) are predicted only by YAGM. We then check the biological plausibility of our novel predictions by using the gene description content in SGD database. Seven (the gene names with red colors) of the nine newly predicted associated genes are known to be involved in cell wall process or glucan biosynthesis just like the query gene *FKS1*, suggesting that YAGM can predict biologically plausible associated genes of a query gene. That is, YAGM can provide alternative candidates of biologically plausible associated genes for biologists to do further experimental investigation.

Second, using *FKS1 *as a query gene, we compare the three lists of top ten associated genes obtained from these three tools when all biological associations are used together. Note that we can only use a single query gene as an example to do the comparison because the query results of STRING and GeneMANIA cannot be downloaded for many query genes at once. As shown in Figure 5, one gene (*GSC2*) is predicted as an associated gene of *FKS1 *by all three tools, but the others are unique for a particular tool. For example, five genes (*SLT2, CNA1, CMP2, ROM2 *and *MNN10*) are predicted only by STRING. Five genes (*SEC7, PXL1, AIM44, APL1 *and *SLX4*) are predicted only by GeneMANIA. Nine genes (*GAS1, ECM33, SMI1, CCW12, KRE6, PSA1, EXG1, PFK2 *and *SCW4*) are predicted only by YAGM. Since our YAGM predicts nine novel associated genes of *FKS1*, we would like check the biological plausibility of our novel predictions by using the gene description content in SGD database [15]. Seven (*GAS1, SMI1, CCW12, KRE6, PSA1, EXG1 *and *SCW4*) of the nine newly predicted associated genes are known to be involved in cell wall process or glucan biosynthesis just like the query gene *FKS1*, suggesting that YAGM can predict biologically plausible associated genes of a query gene. That is, YAGM can provide alternative candidates of biologically plausible associated genes for biologists to do further experimental investigation.

## Conclusions

In this study, we presented the YAGM which provides eight biological associations (including TF binding association, TF regulation association, mutant phenotype association, functional annotation association, physical interaction association, genetic interaction association, and literature evidence association, and expression profile association) for mining associated genes of a query gene in yeast. Among the eight biological associations constructed in YAGM, the first three (TF binding association, TF regulation association, and mutant phenotype association) are novel ones. By comparing the query results of two well-known web tools (STRING and GeneMANIA), we found that YAGM can find out a distinct list of associated genes of a query gene. That is, YAGM can provide alternative candidates of associated genes for biologists to do further experimental investigation. We believe that YAGM will be a useful web tool for yeast biologists. YAGM will be regularly updated based on the newly published literature and the latest release of the YEASTRACT, SGD, BioGRID, and SPELL databases.

## Availability and requirements

YAGM is available at http://cosbi3.ee.ncku.edu.tw/yagm/. The normalized association scores of the eight biological associations between the query gene and every other gene in the yeast genome could be easily downloaded. JavaScript functioning should be enabled in the user-side browsers and the Adobe Flash Player for specific browsers should also be installed. The web interface of YAGM is fully tested on popular browsers: Microsoft IE9, Google Chrome, Apple Safari and Mozilla Firefox. Users are recommended to use these popular browsers for full functionality of YAGM.

## Competing interests

The authors declare that they have no competing interests.

## Authors' contributions

WSW conceived the research topic, collected the original datasets, and provided the essential guidance. WSW and CCW wrote the manuscript. CCW constructed the eight biological similarities and the website of YAGM. MJJ and YCW helped to revise the manuscript and the website. All authors have read and approved the final manuscript.

## Supplementary Material

Additional file 1 **The details of the investigation of the relationships between different biological associations**. Click here for file

## Declarations

The publication of this paper was funded by National Cheng Kung University and Ministry of Science and Technology of Taiwan MOST-103-2221-E-006 -174 -MY2.

This article has been published as part of *BMC Systems Biology *Volume 9 Supplement 6, 2015: Joint 26th Genome Informatics Workshop and 14th International Conference on Bioinformatics: Systems biology. The full contents of the supplement are available online at http://www.biomedcentral.com/bmcsystbiol/supplements/9/S6.
