# Supplementary material for: YAGM: a web tool for mining associated genes in yeast based on diverse biological associations
Source: BMC Syst Biol. 2015 Dec 9;9(Suppl 6):S1. doi: 10.1186/1752-0509-9-S6-S1 (PMC4674844; doi:10.1186/1752-0509-9-S6-S1)
Supplement: Additional file 1 — The details of the investigation of the relationships between different biological associations. [file 1752-0509-9-S6-S1-S1.pdf]

Investigation of the relationships between different biological associations

In order to see how well the different biological associations correlate, for each query gene, we compared the two lists of top 50 associated genes using two different biological associations, respectively. The same process was done for all 6576 possible query genes. Then the average overlap and standard error could be computed.

	PI	GI	TFB	TFR	EP	FA	LE	MP
PI	-	(9.0,0.21)	(3.4,0.13)	(3.4,0.13)	(3.2,0.08)	(11.1,0.21)	(6.6,0.12)	(4.1,0.14)
GI	(9.0,0.21)	-	(3.2,0.14)	(3.2,0.14)	(1.8,0.06)	(10.5,0.22)	(4.4,0.08)	(5.1,0.16)
TFB	(3.4,0.13)	(3.2,0.14)	-	(46.9,0.04)	(2.0,0.06)	(3.8,0.14)	(1.4,0.04)	(2.5,0.12)
TFR	(3.4,0.13)	(3.2,0.14)	(46.9,0.04)	-	(2.0,0.06)	(3.8,0.14)	(1.4,0.04)	(2.5,0.12)
EP	(3.2,0.08)	(1.8,0.06)	(2.0,0.06)	(2.0,0.06)	-	(3.3,0.08)	(4.9,0.08)	(1.8,0.06)
FA	(11.1,0.21)	(10.5,0.22)	(3.8,0.14)	(3.8,0.14)	(3.3,0.08)	-	(8.4,0.11)	(5.0,0.15)
LE	(6.6,0.12)	(4.4,0.08)	(1.4,0.04)	(1.4,0.04)	(4.9,0.08)	(8.4,0.11)	-	(3.2,0.06)
MP	(4.1,0.14)	(5.1,0.16)	(2.5,0.12)	(2.5,0.12)	(1.8,0.06)	(5.0,0.15)	(3.2,0.06)	-

We found that the two lists of top 50 associated genes using two different biological associations have low overlap most of the time, indicating different biological associations are usually lowly correlated. The only exception is the TFB-TFR pair. These two biological associations are highly correlated.

Moreover, in order to know which biological associations are more related to the OAS (overall association score) than the others, for each query gene, we compared the two lists of top 50 associated genes using all eight biological associations together and only one biological association, respectively. The same process was done for all 6576 possible query genes. Then the average overlap and standard error could be computed.

$L_{all} \cap L_{PI}$	$L_{all} \cap L_{GI}$	$L_{all} \cap L_{TFB}$	$L_{all} \cap L_{TFR}$	$L_{all} \cap L_{EPI}$	$L_{all} \cap L_{FA}$	$L_{all} \cap L_{LE}$	$L_{all} \cap L_{MP}$
(8.1,0.10)	(6.5,0.07)	(14.1,0.10)	(14.1,0.10)	(7.0,0.09)	(8.8,0.10)	(14.0,0.12)	(4.7,0.06)

We found that the list of top 50 associated genes using all eight biological associations together have greater average overlap (14 out of 50) with the lists using only TFB association, only TFR association or only LE association than the lists

using the other biological associations. This means that TFB association, TFR association and LE association are more informative than the other associations.
